# Escape of HIV-1 from a Small Molecule CCR5 Inhibitor Is Not Associated with a Fitness Loss

**DOI:** 10.1371/journal.ppat.0030079

**Published:** 2007-06-01

**Authors:** Cleo G Anastassopoulou, Andre J Marozsan, Alexandre Matet, Amy D Snyder, Eric J Arts, Shawn E Kuhmann, John P Moore

**Affiliations:** 1 Department of Microbiology and Immunology, Weill Medical College of Cornell University, New York, New York, United States of America; 2 Division of Infectious Diseases, Department of Medicine, Case Western Reserve University, Cleveland, Ohio, United States of America; National Institute of Allergy and Infectious Diseases, United States of Americ

## Abstract

Fitness is a parameter used to quantify how well an organism adapts to its environment; in the present study, fitness is a measure of how well strains of human immunodeficiency virus type 1 (HIV-1) replicate in tissue culture. When HIV-1 develops resistance in vitro or in vivo to antiretroviral drugs such as reverse transcriptase or protease inhibitors, its fitness is often impaired. Here, we have investigated whether the development of resistance in vitro to a small molecule CCR5 inhibitor, AD101, has an associated fitness cost. To do this, we developed a growth-competition assay involving dual infections with molecularly cloned viruses that are essentially isogenic outside the *env* genes under study. Real-time TaqMan quantitative PCR (QPCR) was used to quantify each competing virus individually via probes specific to different, phenotypically silent target sequences engineered within their *vif* genes. Head-to-head competition assays of *env* clones derived from the AD101 escape mutant isolate, the inhibitor-sensitive parental virus, and a passage control virus showed that AD101 resistance was not associated with a fitness loss. This observation is consistent with the retention of the resistant phenotype when the escape mutant was cultured for a total of 20 passages in the absence of the selecting compound. Amino acid substitutions in the V3 region of gp120 that confer complete AD101 resistance cause a fitness loss when introduced into an AD101-sensitive, parental clone; however, in the resistant isolate, changes elsewhere in *env* that occurred prior to the substitutions within V3 appear to compensate for the adverse effect of the V3 changes on replicative capacity. These in vitro studies may have implications for the development and management of resistance to other CCR5 inhibitors that are being evaluated clinically for the treatment of HIV-1 infection.

## Author Summary

When human immunodeficiency virus type 1 (HIV-1) develops resistance in vitro or in vivo to antiretroviral drugs such as reverse transcriptase or protease inhibitors, its replicative fitness is often impaired (i.e., it grows at a lower rate or to a lesser extent than the parental, inhibitor-sensitive virus). Here, we investigated whether resistance development in vitro to a new class of antiretroviral drugs, the CCR5 inhibitors, has an associated fitness cost. These inhibitors, exemplified by the AD101 compound, are small molecules that bind CCR5, a cell surface protein that HIV-1 uses as a co-receptor during the process of cellular entry. We performed direct-competition assays using sequence-labeled, clonal viruses derived from an AD101 escape mutant, the AD101-sensitive parental isolate, and a passage control isolate, and found that AD101 resistance was not associated with a fitness loss. Furthermore, when the escape mutant was cultured for 20 passages without AD101, it remained resistant. Specific amino acid substitutions conferring AD101 resistance did cause a fitness loss when experimentally introduced into a sensitive clone, but in the naturally selected escape mutant they are probably compensated for by other changes. This work may help understand the development and management of resistance to CCR5 inhibitors now being evaluated clinically to treat HIV-1 infection.

## Introduction

The relative replication ability (fitness) of a human immunodeficiency virus type 1 (HIV-1) quasispecies is governed by how individual clones fluctuate in dominance as they adapt to the host environment [1]. The relative fitness of two viruses in vitro is best estimated by head-to-head competition experiments [2]. Additional selection pressures (e.g., immune responses) influence HIV-1 replication in vivo, but the fitness of HIV-1 in peripheral blood mononuclear cell (PBMC) cultures increased with the extent of viral diversity within a cohort of infected people, and isolates from long-term non-progressors were less fit than ones from rapid progressors [3]. A structured treatment interruption clinical trial showed that HIV-1 fitness also influences the magnitude of viremia rebound and the set point [4].

When HIV-1 develops resistance to the reverse transcriptase and protease inhibitors, its fitness is typically impaired [2,5], which helps explain how beneficial effects of therapy can occur even when HIV-1 replication is incompletely suppressed and highly resistant variants are present [6–9]. Resistance to a fusion inhibitor, enfuvirtide (T-20), has been associated with an in vitro fitness reduction in some [10,11], but not all, studies [12,13]. The instability of resistance when T-20 is discontinued suggests that resistance mutations impair fitness in vivo [14].

The CCR5 inhibitors are a new class of compounds for treating HIV-1 infection and include maraviroc (UK-427,857) and vicriviroc (SCH-D), which are now in phase II/III trials. Resistance to these inhibitors, as to any other antiviral agent [15,16], will inevitably develop during therapy. We have generated several CCR5 inhibitor–resistant isolates and clones in cell culture systems [17–19]. Our best-characterized variants were derived from the HIV-1 primary isolate CC1/85 under the selection pressure of AD101, a precursor of vicriviroc. AD101 resistance is conferred by four amino acid substitutions in the gp120 V3 region; the resistant viruses continue to enter primary CD4^+^ T cells via CCR5 by utilizing the AD101-CCR5 complex [17,19,20]. Resistance of CC1/85 to maraviroc occurs via a similar mechanism [21].

We have investigated whether resistance to AD101 in vitro carries a fitness cost by using a new dual-infection growth-competition assay. This system employs molecularly cloned viruses of defined phenotypes that are essentially isogenic outside the *env* genes under study, but which can be quantified individually using TaqMan quantitative PCR (QPCR). We found that the AD101-resistant Envs were no less fit than those from the parental isolate. Accordingly, the escape mutant remained AD101-resistant during 20 passages in culture in the absence of the selecting compound.

## Results

### Replication Kinetics of Clonal, Env-Chimeric AD101-Resistant and AD101-Sensitive Viruses

We generated clonal, Env-chimeric parental and AD101-resistant viruses by a standard method in which the *env* gene of the pNL4–3 infectious molecular clone [22] is replaced by one of interest [17]. We previously used this system to make Env-chimeric viruses containing *env* genes from parental and AD101-resistant variants of the CC1/85 primary isolate; the chimeras possess the co-receptor usage and entry inhibitor sensitivity properties conferred by the inserted *env* gene [17]. We assessed the replication kinetics of a representative clone of each virus by measuring the rate of p24 antigen production in separate cultures of the same PBMC preparations. In the absence of AD101, the wild-type clone CC1/85 cl.7 and the AD101-resistant variant CC101.19 cl.7 replicated at comparable rates and to similar extents (Figure 1). The resistant virus also replicated comparably (~25 ng/ml of p24 after 8 to 9 d) in the presence of 1 μM AD101, a concentration that completely inhibited p24 production by CC1/85 cl.7 (Figure 1). The high-level AD101 resistance of CC101.19 cl.7 was not, therefore, associated with any apparent reduction in replication efficiency. However, comparing replication rates in different mono-infection cultures does not always allow the identification of significant differences in replicative fitness [2,23,24]. Better discrimination can be achieved when both viruses replicate in the same culture. We therefore developed an assay that could accurately quantify the replication of two different clonal, Env-chimeric viruses in the same culture.

**Figure 1:**
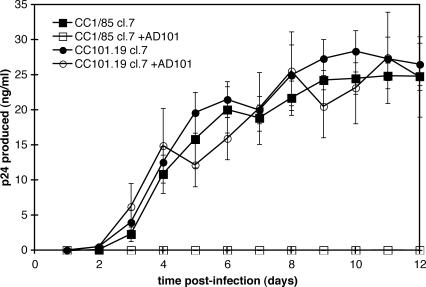
Replication Kinetics of Clonal, Env-Chimeric Viruses with an AD101-Sensitive or an AD101-Resistant *env* Gene The amount of p24 produced from PBMC culture infected with equal titers of CC1/85 cl.7 (squares) or CC101.19 cl.7 (circles), in the absence (filled symbols) or presence (open symbols) of 1 μM AD101, was determined at the indicated time points. Duplicate wells were used to derive each experimental value in each experiment, and the data shown are the average ± standard error of the mean (SEM) of values from five independent experiments.

### Development and Validation of a TaqMan QPCR–Based Replicative Fitness Assay

The main challenge in determining the outcome of dual-infection growth-competition experiments is to devise a strategy to detect the two competing viruses without introducing any changes in other regions of the HIV-1 genome that could potentially affect fitness. We therefore created sequence tags for virus detection by altering 11 nucleotides between positions 5466 and 5493 of pNL4–3, a region containing only the *vif* open reading frame. The changes create a stretch of synonymous mutations that do not affect the Vif protein sequence, do not overlap the central termination sequence [25], and do not significantly interfere with RNA secondary structure. Probes were designed for specific annealing to either the wild-type (vifX) or the silent-mutated (vifY) *vif* sequences under the conditions of a TaqMan QPCR assay (Figure 2A). A standard plasmid carrying tandem copies of both the vifX and vifY *vif* sequences was constructed to serve as a template for standardizing QPCR results when both TaqMan probes target their specific sequences. Seven 10-fold serial dilutions of a known molar concentration of this plasmid were used to generate standard curves in every QPCR experiment. The relationship between the plasmid copy number and the QPCR threshold cycle (*C_T_*) value was log-linear, and the plasmid standard was efficiently detected, the sensitivity limit being <50 copies of plasmid DNA per reaction (Figure 2B; unpublished data). The average PCR efficiency for detection of the vifX and the vifY sequences was 91% and 95% for the six representative vifX and vifY multiplexed assays shown in Figure 2B, respectively.

**Figure 2:**
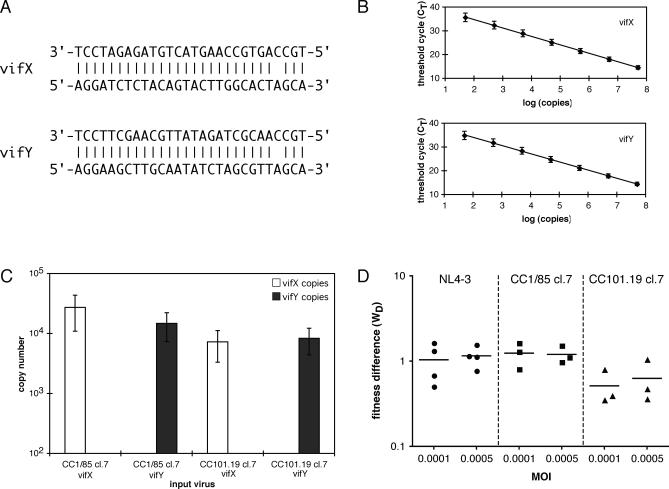
The Use of the TaqMan QPCR Assay to Determine Replicative Fitness (A) The sequences (3′ to 5′) of the vifX and vifY TaqMan probes are shown on top, the sequences of the vifX and vifY *vif* alleles are shown below. Each probe differs from its target sequence by one nucleotide because the vifX probe was designed to match the consensus for HIV-1 subtype B *vif* in this region. Each probe binds to the sense strand of proviral DNA. The probes are aligned to the target sequences with bars indicating positions of identity. The two probes and the two *vif* alleles differ at 11 nucleotides each. The vifY *vif* allele that was engineered to tag the reference viruses contains only synonymous changes. (B) The plot demonstrates the working range and reproducibility of the QPCR assay. The average threshold cycle (*C_T_*) values obtained in six representative multiplexed assays are shown ± standard deviation (SD). In each assay, seven serial dilutions of the standard plasmid ranging from 5 × 10^7^ to 5 × 10^1^ DNA templates were measured using TaqMan probes specific either for the vifX (Cy5 fluorescence, top panel), or the vifY sequence (FAM fluorescence, bottom panel). The correlation coefficients (*R* ^2^) of the two standard curves were >0.995 and the PCR efficiencies were >90%. (C) The specificity of the QPCR assay is depicted. Four independent PBMC cultures were singly infected with one of the CC1/85 cl.7 or the CC101.19 cl.7 viruses containing either the vifX or the vifY *vif* sequence. Genomic DNA from each culture was PCR amplified and then used in the multiplexed TaqMan QPCR assay, as described in Materials and Methods. The average number of copies of each *vif* allele detected per QPCR reaction for each of the four mono-infections from six representative experiments is shown ± SEM. The lower limit for copy number is set to 100 for plotting purposes, although 50 copies can be quantified reproducibly, and the allele that was not present in the infection was never detected in any quantifiable amount in singly infected cultures. (D) The *vif* tag has no effect on replicative fitness. Competitive replication assays were performed in which viruses bearing the NL4–3 (circles), CC1/85 cl.7 (squares), or CC101.19 cl.7 (triangles) *env* genes and the vifX *vif* allele were competed against viruses bearing the same *env* genes but the vifY sequence. The fitness differences (W_D_) at each indicated MOI were calculated as described in Materials and Methods. The calculated fitness differences of each vifX virus relative to the vifY virus in each experiment are depicted, with the bar showing the mean value of three or four independent experiments. A fitness difference of 1 arises when the two competing viruses are of equal replicative fitness; a value < 1 indicates that the virus with vifY had the greater replicative fitness, > 1 that the virus with vifX was fitter.

We inserted the *env* genes of the parental clone CC1/85 cl.7 and its AD101-resistant variant CC101.19 cl.7 into the vifY version of pNL4–3 (Table 1). These vifY-containing clones were used as references against which all the vifX-containing viruses were competed. We always report fitness differences in terms of the replication of the vifX virus relative to the vifY reference. A plasmid expressing NL4–3 *env* and containing the vifY sequence was also made to serve as a reference virus in some experiments (Table 1).

**Table 1:**
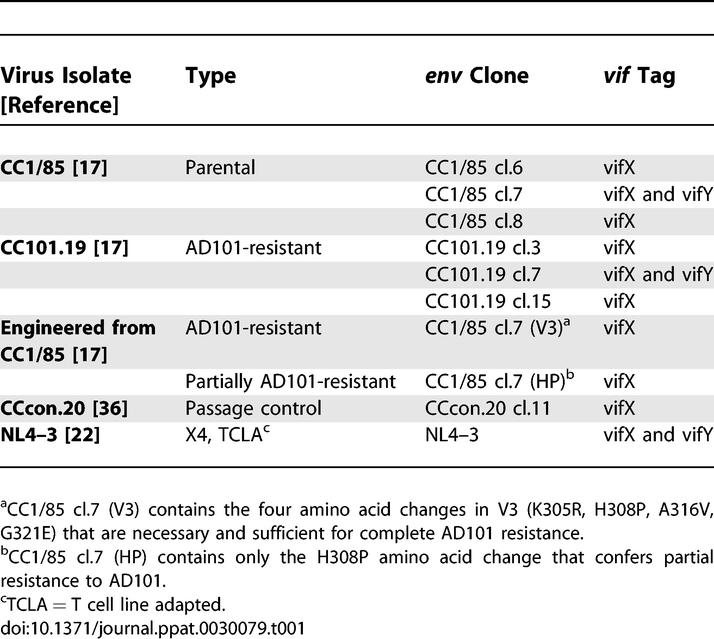
Nomenclature and Properties of Viruses Used in This Study

We tested the assay's specificity by using the vifX and vifY probes to simultaneously detect their target sequences in DNA amplified from cells infected with only one virus. Separate PBMC cultures were infected with either the vifX or vifY versions of the CC1/85 cl.7 or CC101.19 cl.7 clones, and the vifX and vifY sequences were detected simultaneously using the multiplexed QPCR assay (Figure 2C). Only the *vif* allele present in the virus used to inoculate each culture was ever detected; the average DNA copy number per QPCR reaction derived from the cognate probe was typically >10^4^, but from the non-cognate probe, <50 (Figure 2C). We routinely use single-infection cultures as specificity controls and have never detected the *vif* allele not known to be present in these cultures (unpublished data).

In each set of competition assays, the two viruses were added to PBMC cultures at the same multiplicity of infection (MOI), 0.0001. In an otherwise identical culture, each virus was also added at a higher MOI, 0.0005. In additional cultures, one virus was present at an MOI of either 0.0001 or 0.0005, the other in 10-fold excess. The resulting average copy numbers detected per QPCR reaction were then used to determine the proportion of each virus present in each test culture on day 10 (NL4–3) or day 14 (CC1/85 cl.7 or CC101.19 cl.7) (see Materials and Methods). The relative proportions of each virus at each condition were determined and the fitness difference (W_D_) value was calculated for each MOI, as described elsewhere [3]. Under these experimental conditions, W_D_ is an approximation of the fold difference in the replication ability of the two viruses added to a dual-infection culture. The W_D_ value is reported as the fitness of the virus bearing the vifX allele relative to a reference virus containing vifY.

To determine if the *vif* tag could influence viral fitness and affect the outcome of head-to-head competitions in dual-infection cultures, we established competitions with two viruses with the same Env protein (NL4–3, CC1/85 cl.7 or CC101.19 cl.7) but a different *vif* allele (i.e., vifX or vifY). The W_D_ values were always ~1; that is, there was no consistent pattern of victory or defeat (Figure 2D). In addition, when *vif* was sequenced from cultures mono-infected for 10 d with the modified (vifY) version of NL4–3, it was unchanged (unpublished data). Hence, as intended, the *vif* tag does not affect fitness. The extent of assay-to-assay variation in this type of experiment also indicates that W_D_ values differing by <3-fold are not meaningful (Figure 2D; unpublished data); conversely, W_D_ values >3 or <0.33 indicate that two viruses present in direct-competition assays have different replication capacities.

Hence, we have developed a sensitive and specific assay to quantify the replication of two different clonal viruses in the same growth-competition culture.

### Replication and Replicative Fitness of AD101-Resistant and AD101-Sensitive Clonal Viruses

We used the assay to determine whether there was a difference in replicative fitness between AD101-resistant and AD101-sensitive viruses. We first measured the replication of R5 clonal, Env-chimeric viruses (Table 1) in PBMC mono-cultures for 14 d, using the TaqMan assay as an endpoint (Figure 3). Every virus yielded a reproducible copy number when tested at an MOI of 0.0005. All three AD101-sensitive, parental CC1/85 clones were inhibited when 1 μM AD101 was added 1 h prior to infection, the average copy number reduction ranging from 33- to 480-fold for the different clones. The AD101-sensitive CCcon.20 cl.11 passage control clone was also strongly inhibited by AD101, with a 990-fold reduction in copy number.

**Figure 3:**
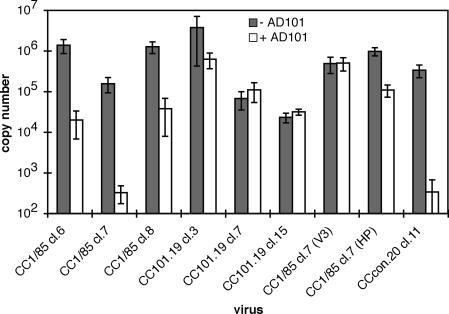
Replication of Clonal, Env-Chimeric Viruses in Singly Infected Cultures The extent of replication is shown as the mean copy number per QPCR reaction ± SEM from three to 47 independent, singly infected cultures. AD101 was absent (shaded bars) or was present at 1 μM (open bars). The initial MOI was 0.0005.

In contrast, the clones from the CC101.19 isolate were AD101 resistant; there were no significant differences in copy numbers between the AD101-treated and control cultures, even at inhibitor concentrations of 20 μM, and at either MOI (Figure 3; unpublished data). The experimentally mutated clone CC1/85 cl.7 (V3), which contains four amino acid changes in V3 conferring complete AD101 resistance [17], was also strongly AD101 resistant (Figure 3). The CC1/85 cl.7 (HP) clone was inhibited by AD101, but the copy number reduction was only 9-fold, substantially less than the 480-fold reduction seen with the parental CC1/85 clone from which CC1/85 cl.7 (HP) was derived by introducing the H308P substitution. The H308P polymorphism, also present in CC1/85 cl.8, was very rapidly selected for by AD101 and confers partial resistance in a conventional, PBMC-based assay (i.e., a 5- to 10-fold shift in IC_50_) [17]. Thus, the pattern of resistance of the various clones in the QPCR-based assay qualitatively reproduces the phenotypes the infectious, replication-competent viruses display in a standard virus production assay [17].

Variations in how viruses replicate in single-infection cultures are not always true indications of replicative fitness differences [2,23,24]. We therefore performed competitive fitness assays in dual-infected cultures. Each of the clonal viruses listed in Table 1 that bore a vifX allele was tested in a competitive dual-infection experiment against the vifY-bearing versions of both the AD101-sensitive virus CC1/85 cl.7 and the AD101-resistant virus CC101.19 cl.7. The competition cultures were again established at two MOIs (0.0001 and 0.0005) and at three ratios at each MOI (1:1, 1:10, 10:1). The fractional proportions of each virus present in each culture were calculated, with the averages over multiple experiments used to calculate W_D_ values for the two test viruses at each MOI [3].

In general, the AD101-sensitive clone CC1/85 cl.6 and the engineered, partially AD101-resistant clone CC1/85 cl.7 (HP) substantially out-competed each reference virus, replicating 10- to 86-fold better in the dual-infection cultures (Figure 4A and 4B). In contrast, the AD101-resistant clone CC101.19 cl.3 and the engineered, AD101-resistant clone CC1/85 cl.7 (V3) were consistently less fit than either reference virus, replicating 2.4- to 71-fold less well under all the various conditions described in Figure 4A and 4B. CC1/85 cl.7 (V3) did not replicate detectably in the presence of the CC101.19 cl.7 reference virus in any experiment, so no W_D_ could be calculated for this pairing; however, based on the known parameters of the assay, the inference is that CC1/85 cl.7 (V3) is >1,000-fold less fit than CC101.19 cl.7. The relative fitness of the other AD101-resistant and AD101-sensitive clones all fell within a spectrum, with some patterns evident. For example, the AD101-sensitive clone CC1/85 cl.8 and the AD101-resistant clones CC101.19 cl.7 and CC101.19 cl.15 were generally fitter than CC1/85 cl.7 (parental) and CCcon.20 cl.11 (passage control) (Figure 4A and 4B). There was no correlation between the copy numbers produced after 14 d in single-infection cultures and the W_D_ values determined from dual-competition cultures (compare Figure 3 with Figure 4A and 4B). The paradoxical nature of this finding reinforces the unreliability of judging HIV-1 fitness based solely on single-infection cultures [2,23,24].

**Figure 4:**
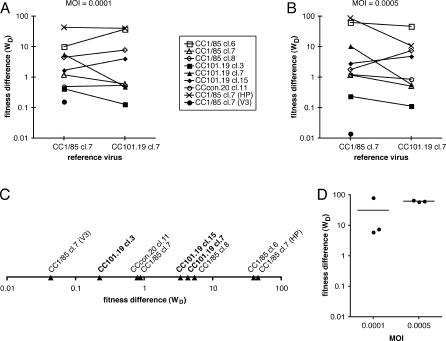
Replicative Fitness of the Clonal Env-Chimeric Viruses Relative to the CC1/85 cl.7 and CC101.19 cl.7 Reference Viruses (A and B) The replicative fitness of each vifX clonal virus relative to the indicated vifY reference virus was calculated from the mean virus proportions from three to seven independent competition experiments, and is shown for MOIs of (A) 0.0001 and (B) 0.0005. Fitness differences < 1 indicate the vifX virus is less fit than the vifY reference virus, > 1 that the vifX virus is the more fit. The vifX viruses used were: CC1/85 cl.6 (open squares), CC1/85 cl.7 (open triangles), CC1/85 cl.8 (open diamonds), CC101.19 cl.3 (filled squares), CC101.19 cl.7 (filled triangles), CC101.19 cl.15 (filled diamonds), CCcon.20 cl.11 (open circles), CC1/85 cl.7 (HP) (X symbol), and CC1/85 cl.7 (V3) (filled circles). (C) The plotted fitness differences were averaged between reference viruses and MOIs from (A) and (B). Thus, the fitness differences shown are all relative to an arbitrary reference point (= 1.0). The three clones from the AD101-resistant CC101.19 isolate are shown in bold (C). (D) Independent fitness difference determinations generate reproducible results. The W_D_ values for CC1/85 cl.6 vifX relative to CC1/85 cl.7 vifY are plotted for three individual experiments at the MOIs indicated. This pairing was chosen because of the large difference in fitness between them. The W_D_ values were determined from the proportion of each virus within a single experiment. The bars represent the means of these W_D_ values (31 and 63 at MOIs of 0.0001 and 0.0005, respectively). The same data were analyzed in (A) and (B) by taking the average of the proportions between the three experiments before calculating the W_D_, thereby minimizing the influence of outliers. The W_D_ values derived from (A) and (B) were 10 and 63 at MOIs of 0.0001 and 0.0005, respectively.

Because similar patterns of relative fitness differences were seen regardless of the reference virus or MOI, we averaged the fitness differences derived for each test virus in the various individual cultures to derive a replicative fitness rank order relative to an arbitrary reference point (Figure 4C). The resulting fitness differences spanned an ~1,050-fold range; the two extremes represent viruses engineered from CC1/85 cl.7 to contain either one (CC1/85 cl.7 (HP), the most fit) or four amino acid changes in V3 (CC1/85 cl.7 (V3), the least fit). The fitness spectra of the various naturally occurring AD101-sensitive and AD101-resistant clones overlapped within this range.

In theory, at high MOIs, recombination between a vifX and a vifY virus with the site of recombination between the *vif* gene and the *env* gene could confound our results. If this happened, competitions in which the observed W_D_ value was very high or very low would be poorly reproducible. We derived the data in Figure 4A and 4B by determining the average relative virus proportions from at least three experiments before calculating the W_D_ value, thereby minimizing the influence of outliers. In Figure 4D we reanalyzed the data for one set of competitions (CC1/85 cl.6 vifX versus CC1/85 cl.7 vifY) by calculating the W_D_ values within each individual experiment. The average W_D_ values shown in Figure 4D were 31 and 63 at MOIs of 0.0001 and 0.0005, respectively, and the W_D_ values derived from Figure 4A and 4B were 10 and 63 at these respective MOIs. The similarity in outcome between the two approaches suggests that outliers do not contribute significantly to the observed fitness differences, implying that recombination is not occurring in the cultures.

### Replication and Replicative Fitness of AD101-Resistant and AD101-Sensitive Viral Isolates

The above experiments suggest that AD101 resistance is not necessarily associated with a replicative fitness reduction (Figure 4). However, all of these data sets were generated with clonal, recombinant viruses that are identical outside *env,* and not with the original uncloned isolates that emerged in the resistance-selection experiments. Any analysis of these isolates is complicated by the likelihood that additional sequence changes occurred outside *env* while HIV-1 adapted to prolonged culture in PBMCs. Although we thought it unlikely that clonal bias had obscured any underlying fitness differences, we tested the original resistant isolates to gain further insights into the relative fitness of the AD101 escape mutants.

The copy numbers obtained for each isolate after 14 d of PBMC culture were similar when the vifX probe was used (Figure 5A), but no copies were detectable with the vifY probe (unpublished data). Thus, these isolates can be used as test viruses in competitions against vifY-tagged references. As expected, replication of the CC1/85 isolate, but not the CC101.19 isolate, was impaired by 1 μM AD101 (Figure 5A). The average copy numbers calculated for both isolates were somewhat lower than those seen with the corresponding clones under similar conditions (compare Figure 5A to Figure 3). This is not surprising since the latter are based on the genomic backbone of NL4–3, a clone adapted for optimal growth in cell culture [22].

**Figure 5:**
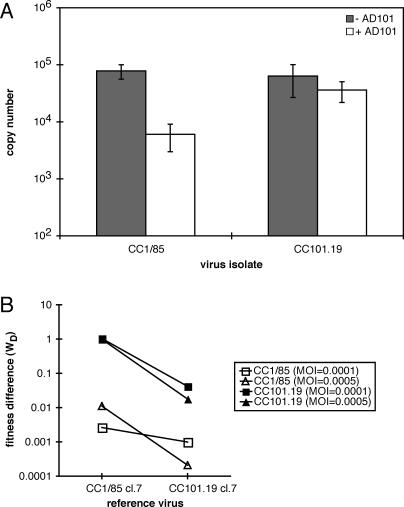
Replication and Replicative Fitness of the CC1/85 and CC101.19 Isolates (A) PBMC cultures were singly infected with the indicated isolates at an MOI of 0.0005 in the presence (open bars) or absence (shaded bars) of 1 μM AD101. The vifX *vif* probe was used in the TaqMan QPCR assay to derive the copy number per QPCR reaction; the values shown are the means ± SEM from three independent experiments. (B) The fitness differences of the isolates CC1/85 (open symbols) or CC101.19 (filled symbols) as detected by the vifX probe are shown relative to the indicated vifY reference viruses at MOIs of 0.0001 (squares) or 0.0005 (triangles). The values are derived from the mean virus proportions from three independent experiments.

Having shown that the QPCR assay can be used to study the replication of the isolates on which the clones were based, we examined the fitness of the CC1/85 and CC101.19 isolates relative to the vifY-tagged reference clones CC1/85 cl.7 and CC101.19 cl.7 in head-to-head competitions without AD101. The outcome of these assays allowed us to determine the comparative fitness of the two isolates. The replicative fitness of each isolate was always less than that of the corresponding reference clone (W_D_ < 1, Figure 5B). This is likely to be a consequence of using pNL4–3 as a background vector into which *env* genes are inserted to make chimeric viruses. NL4–3 has probably acquired several mutations outside *env* that improve its fitness in vitro, compared to primary isolates like CC1/85 [22]. When CC1/85 cl.7 was used as the reference virus, the AD101-resistant isolate CC101.19 was found to be 89- and 390-fold more fit than the parental CC1/85 isolate at the MOIs of 0.0005 and 0.0001, respectively. With CC101.19 cl.7 as reference, CC101.19 was 85- and 42-fold more fit than CC1/85 at the same two MOIs. The average of these four fitness difference estimates is ~150. Hence, if any sequence changes outside *env* did arise during culture of CC101.19, they improved its fitness. A conservative conclusion is that the development of AD101 resistance by CC101.19 has not come at the price of a dramatic fitness loss.

### Stability of the AD101-Resistant Phenotype in the Absence of the Inhibitor

If the acquisition of AD101 resistance caused a replicative fitness loss in PBMC cultures, the resulting AD101-resistant isolate would revert to sensitivity when the cultures were continued without the inhibitor. Because the above studies, using both clones and isolates, implied that AD101 resistance did not create an unfit virus, we hypothesized that any such reversion would be slow, and might not even occur. We reported that when the AD101-resistant isolate generated after 22 passages in the presence of AD101 (CC101.22) was cultured in PBMCs without AD101 for nine additional passages (CC101.22R9), it remained highly AD101 resistant [19]. We therefore returned the CC101.22R9 isolate to culture in PBMCs for 11 additional passages, hence 20 in total, without AD101. The AD101 sensitivity profiles of the CC1/85, CC101.19, and CC101.22R9 isolates, and ones receiving 10, 15, or 20 passages without AD101 (CC101.22R10, CC101.22R15, and CC101.22R20, respectively), were determined (Figure 6). All the isolates from the AD101-free culture remained completely resistant to AD101 concentrations as high as 5 μM, whereas the parental CC1/85 isolate was inhibited in the 1–10 nM range. Hence the AD101-resistant phenotype is highly stable, which is consistent with the conclusion that it is not associated with any significant decrease in replicative fitness in PBMC cultures.

**Figure 6:**
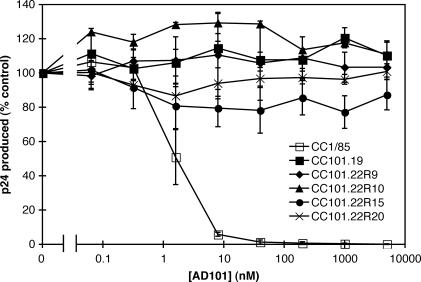
AD101 Sensitivity of AD101-Resistant Isolates after Extensive Passage in the Absence of AD101 PBMC cultures were infected with the parental isolate CC1/85 (open squares), the resistant isolate CC101.19 (filled squares), or isolates CC101.22R9 (filled diamonds), CC101.22R10 (filled triangles), CC101.22R15 (filled circles), and CC101.22R20 (X symbol) from the reversion cultures, in the presence of different concentrations of AD101. The amount of p24 produced in each AD101-containing culture after 7 d is shown as a percentage of that produced by the same isolate in the absence of inhibitor. The values shown are the means of four independent experiments ± SEM.

### The Effect of AD101 on the Fitness of AD101-Resistant Clonal Viruses

Finally, we sought to determine whether AD101 affected the replicative fitness of AD101-resistant viruses; these viruses are clearly drug-resistant and not drug-dependent, but does a dose of the drug give them a boost? The standard QPCR assay system cannot be used to determine the relative fitness of a single virus in the presence and absence of an inhibitor. Instead, we used a vifY-tagged version of the X4 clone NL4–3 as a reference virus that is unaffected by AD101, a CCR5 inhibitor, because it uses CXCR4 for entry. The vifY-tagged NL4–3 virus was competed against the AD101-resistant vifX-tagged CC101.19 cl.7 clone in cultures either lacking or containing AD101 at a concentration (20 μM) that saturates CCR5 (Figure 7A). Because NL4–3 replicates very efficiently in PBMC culture, we added AMD3100 (20 nM), a CXCR4 inhibitor specific to this virus (i.e., one that would not affect the R5 virus CC101.19 cl.7), to reduce its replication rate to a level comparable to that of its competitor in the dual-infection cultures. This AMD3100 concentration had no effect on the copy numbers of CC101.19 cl.7 determined in single-infection cultures, nor did AD101 affect NL4–3 replication (unpublished data).

**Figure 7:**
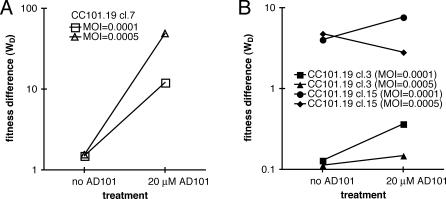
The Effect of AD101 on the Fitness of Clonal, Env-Chimeric AD101-Resistant Viruses (A) Replicative fitness differences in PBMCs after 14 d of culture were determined at MOIs of 0.0001 (open squares) or 0.0005 (open triangles) for the vifX CC101.19 cl.7 virus relative to the vifY NL4–3 reference virus, in the presence of 20 nM AMD3100 and in the presence or absence of 20 μM AD101, as indicated. (B) Replicative fitness differences in PBMCs after 14 d of culture were determined at MOIs of 0.0001 (squares, circles) or 0.0005 (triangles, diamonds) for the vifX CC101.19 cl.3 virus (squares, triangles) or the vifX CC101.19 cl.15 virus (circles, diamonds), relative to the vifY CC101.19 cl.7 reference virus, and in the presence or absence of 20 μM AD101, as indicated. In both panels, the plotted values are derived from the mean virus proportions from three independent experiments.

Under these conditions, in the absence of AD101, the fitness of CC101.19 cl.7 was indistinguishable (W_D_ ~1.5) from that of NL4–3 at either MOI, indicating that AMD3100 had indeed adjusted the replication rate of NL4–3 to the intended degree (Figure 7A; unpublished data). In the presence of 20 μM AD101, the corresponding fitness differences were 12 and 49 at MOIs of 0.0001 and 0.0005. Thus, at these MOIs, CC101.19 cl.7 is 8.1- to 32-fold fitter when AD101 (20 μM) is present (Table 2); AD101 therefore modestly enhances the replicative fitness of the CC101.19 cl.7 clone.

**Table 2:**
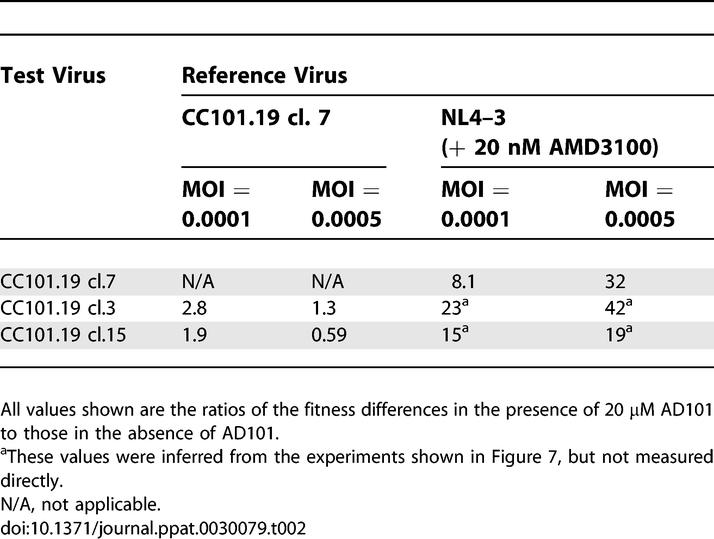
Effect of 20 μM AD101 on the Fitness Differences of AD101-Resistant Test Viruses Relative to the Indicated Reference Viruses

To see whether the enhancing effect of AD101 was unique to CC101.19 cl.7, dual-infection experiments were performed in which the vifX *vif*-bearing clones CC101.19 cl.3 and CC101.19 cl.15 were competed against vifY-tagged CC101.19 cl.7, with AD101 again either absent or present at 20 μM. We showed above that CC101.19 cl.15 was fitter than CC101.19 cl.7 in the absence of AD101, whereas CC101.19 cl.3 was less fit (Figure 4A and 4B). When AD101 was present, the existing fitness differences between CC101.19 cl.15 or CC101.19 cl.3 and CC101.19 cl.7 were only minimally altered. For example, the fitness of CC101.19 cl.15 relative to CC101.19 cl.7 was increased by 1.9-fold at an MOI of 0.0001, but at an MOI of 0.0005 it was decreased by 1.7-fold; such changes are insignificant (Figure 7B; Table 2). Similarly, when AD101 was present, the fitness of CC101.19 cl.3 relative to the CC101.19 cl.7 reference virus was increased by 2.8- and 1.3-fold at the low and high MOIs. The AD101-resistant CC1/85 cl.7 (V3) virus was found not to replicate detectably when in competition with the CC101.19 cl.7 reference virus, whether AD101 was present or not; no fitness differences could therefore be calculated for this pairing (unpublished data).

Overall, we conclude that AD101 did not significantly affect pre-existing fitness differences between the various CC101.19-derived clones. A corollary is that, like CC101.19 cl.7, the fitness of the AD101-resistant clones CC101.19 cl.15 or CC101.19 cl.3 is also increased when AD101 is present during a 14-d PBMC culture (summarized in Table 2). Thus, all the AD101-resistant viruses we tested are not just resistant to the selecting compound, they actually replicate more efficiently in its presence, albeit only to a modest extent that may not always be apparent in conventional PBMC assays.

## Discussion

How HIV-1 adapts to its environment can be quantified by measuring its ability to produce infectious progeny (fitness). Fitness determinations can help us understand how HIV-1 adapts, either in vitro or in vivo, to selection pressures imposed by antiretroviral drugs. Strains resistant to reverse transcriptase or protease inhibitors are often less fit than their parents (reviewed in [2,5]). Similarly, resistance to enfuvirtide is often associated with reduced fitness in vitro or in vivo in some [10,11,14,26,27], but not all [13], studies. Resistance to the CXCR4 inhibitor AMD3100 in vitro also incurs a fitness cost [28]. We have been studying resistance to small molecule CCR5 inhibitors in vitro, exemplified by AD101, a vicriviroc precursor whose development was discontinued for pharmocokinetic reasons [17–20]. Here, we have investigated the fitness cost of AD101 resistance, developing a new assay that may have general utility.

Assays of the in vitro replicative fitness of drug-resistant HIV-1 strains, or phenotypic variants in general, have become increasingly sophisticated [10,13,29,30]. In particular, the use of QPCR technology has simplified the procedure while also improving the detection sensitivity for competing viral variants [29,31–33]. Yet, limitations still exist. For example, not all HIV-1 strains can be analyzed easily, because genetic variation affects the binding sites for standard primers. Assay precision can also be problematic, particularly when fitness differences are slight. And assay procedures cannot affect viral fitness; any probe sequences inserted into the genome must be fitness-neutral.

Our new assay involves detecting two different *vif* alleles in the genomic DNA from PBMCs cultured with viruses tagged with these sequences. The assay can detect as little as a 3-fold fitness difference between two viruses in a dual-competition assay, and the genetic sequences detected by the probes have no effect on fitness (the probes detect wild-type and mutant *vif* gene sequences that have no effect on the Vif protein or other known functions of the genome). The method is flexible: the fitness influence of any gene other than *vif* can be tested by inserting it into the NL4–3 vifY virus and competing the chimera against a relevant NL4–3 vifX clone. This allows the fitness impact of small genetic differences to be assessed, without redesigning probes specific for each new test virus. The method can also work with uncloned isolates, because the vifX probe was designed to recognize a *vif* region highly conserved among subtype B strains; here, we have studied both clones and isolates.

Recombination is a concern with all dual-competition fitness assays, here specifically between the vifX- and vifY-tagged viruses. The likelihood of this occurring probably increases with the distance between the gene under study and the probe-binding sequence in *vif*. We have used MOIs low enough (0.0001 and 0.0005) to render recombination highly improbable, based on previous studies [34,35]. The likelihood of recombination between two viruses of equal fitness is estimated as < 1% per 1,000 nucleotides at an MOI of 0.001 [34]. As we have used even lower MOIs, it is unlikely that enough dual-infected cells are present for recombination to occur detectably. This view is supported by the reproducibility of the fitness differences we measured (see, for example, Figure 4D); frequent recombination would create a much wider scatter of results, with outliers.

We saw no correlation between fitness differences (Figure 4) and the absolute copy numbers produced by the same viruses in single-infection cultures (Figure 3). This reinforces reports that replication rates in single-virus cultures are not appropriate measures of fitness, and that dual-competition assays are the best way to assess the relative fitness of different viruses [2,23,24].

### In Vitro Replicative Fitness of AD101-Resistant and Passage Control *env* Clones

We have shown that an R5 primary isolate, CC1/85, becomes highly resistant when serially passaged in PBMC culture with increasing concentrations of AD101 [19]. A resistant isolate from passage 19, CC101.19, remained CCR5-dependent for replication in PBMC culture. *Env* sequence changes were necessary and sufficient to confer AD101 resistance, which developed in two stages: a single amino acid polymorphism in V3, H308P, was selected during the earliest passages with AD101 and conferred modest (5- to 10-fold) resistance; three de novo mutations, also in V3 (K305R, A316V, G321E), arose later to produce a highly (>20,000-fold) resistant virus [17]. The H308P polymorphism increases the efficiency with which HIV-1 can use low CCR5 levels for entry, whereas the later changes create gp120 proteins that can recognize the inhibitor-bound conformation of CCR5 [17,19,20].

When we commenced this study, several lines of evidence suggested the AD101 escape mutants might not have reduced fitness relative to the parental and passage control isolates: (i) The resistant phenotype as well as the underlying genetic changes were stable, in the short-term, after the inhibitor-selection pressure was withdrawn; (ii) the replication rates of the CC1/85 and CC101.19 clones in primary CD4^+^ T cells were similar; and (iii) the CC101.19 clones did not require AD101 for replication [17,19]. Our initial experiments supported these findings (Figure 1).

We therefore used the dual-competition assay to compare three *env* clones from both the parental CC1/85 and the AD101-resistant CC101.19 isolates in the vifX background, with vifY-tagged reference viruses containing *env* genes from either CC1/85 cl.7 or CC101.19 cl.7. Three other *env* genes inserted into vifX-tagged viruses were similarly studied: a CC1/85 *env* engineered to contain the V3 H308P change (CC1/85 cl.7 (HP)); a derivative of the same *env* containing all four V3 changes that confer complete AD101 resistance (CC1/85 cl.7 (V3)); and an *env* cloned from the 20th-passage control isolate (CCcon.20 cl.11) (Table 1). Using W_D_ values averaged over MOIs and reference viruses, with W_D_ = 1 representing an arbitrary reference point for comparisons, the three *env* clones from CC1/85 spanned an ~50-fold range of fitness differences, as did the three CC101.19 clones. A critical point is that these ranges overlapped each other, implying that AD101 resistance in vitro caused no intrinsic fitness loss (Figure 4C).

The two extremes in the fitness range are represented by the two engineered viruses. The single H308P change allowing CC1/85 cl.7 to use free CCR5 more efficiently confers an ~50-fold fitness increase when AD101 is absent. Despite this, the minor H308P variant did not expand in frequency in the passage control culture, for reasons that have yet to be understood [17,20]. The three additional V3 changes that then permit use of the AD101-bound form of CCR5 produce a virus ~20-fold less fit than the parental CC1/85 cl.7 clone. Compared, then, to the partially resistant single mutant, the three later-arising substitutions responsible for complete resistance cause an ~1,000-fold fitness loss. We note, however, that these engineered viruses are isogenic elsewhere in *env,* whereas the original uncloned isolates and the derivative clones are not. Hence, the fitness loss associated with the three later-arising V3 substitutions may be compensated for by additional changes elsewhere in gp120 (or even in gp41); we previously noted the possible importance of such compensatory changes [17,18]. However, it is unlikely that any compensatory changes arose directly from a selective pressure for increased fitness, because V3 is the only *env* region undergoing selection after the fourth passage with AD101 [17]. Rather, we believe the V3 changes arose in an Env context that was selected for early in the escape process, and in that particular environment they cause no fitness reduction. These scenarios contrast markedly with the fitness effects associated with the pathways to protease and reverse transcriptase inhibitor resistance; there, primary resistance mutations affecting the action of the drug typically confer a fitness loss, but are compensated for by secondary mutations that eventually restore fitness [2].

We passaged a control isolate in PBMCs in the absence of AD101 [36]. The resulting 19th passage isolate, CCcon.19, and a related clone, CCcon.20 cl.11, became more sensitive to soluble CD4 without any change in AD101 sensitivity, due to slowly accumulating sequence changes in the V1/V2 region [36]. We hypothesized that these passage control viruses had mutated to improve their fitness in the absence of counter-selection by the neutralizing antibodies that are present in vivo [36]. We now show that the fitness of CCcon.20 cl.11 falls within the range spanned by the various CC1/85 and CC101.19 clones. As the V1/V2 changes accumulated slowly, any fitness differences they do impart may be too minor to be quantified, and/or that more CCcon.20 *env* clones would need to be studied to detect them. Genetic and phenotypic changes during prolonged cultures of primary isolates in PBMCs have now been described by others [21,37].

### In Vitro Replicative Fitness of the AD101-Resistant Isolates

We have studied the fitness of HIV-1 clones that are identical outside *env,* whereas the uncloned isolates from our original resistance-selection experiments may have had other sequence changes influencing their phenotypes. Analysis of these isolates is complicated by any sequence changes arising as the virus adapts to extended culture in PBMCs, rather than due to the effect of the CCR5 inhibitor. Even so, we considered that studying the isolates could still be informative about gross fitness changes, and might alleviate concerns about whether the limited number of clones available for study creates any clonal bias. Compared to either reference virus (CC1/85 cl.7 or CC101.19 cl.7), the AD101-resistant CC101.19 isolate had, on average, a W_D_ value ~150-fold higher than the parental CC1/85 isolate (Figure 5B). Even though we cannot exclude an influence of the culture process on fitness, it does seem reasonable to conclude that AD101 resistance is not associated with a fitness loss at the level of either the uncloned isolates or the derivative clones.

A corollary of this conclusion is that AD101 resistance should remain stable during extended in vitro passage in the absence of the inhibitor. We showed that CC101.22, a resistant isolate from passage 22 of the original AD101 selection culture, retained its phenotype during nine additional passages in the absence of AD101 [19]. Moreover, the four V3 changes responsible for AD101 resistance also remained stable over this time [17]. We have now cultured this isolate without AD101 for an additional 11 passages, 20 in all, a total comparable with the 22 passages the virus had received with AD101 present. Isolates from these cultures remained highly AD101 resistant (Figure 6), strongly supporting the conclusion that complete resistance to AD101 does not confer a significant fitness loss.

In contrast, another CCR5 inhibitor–resistant isolate, generated under the selection pressure of SCH-C in PM1 cells and derived from the subtype G strain JV1083, did gradually revert to sensitivity when it was passaged for prolonged periods without SCH-C. The genetic pathway to reversion was similar, but not identical, to the pathway to resistance, with V3 changes predominating in both instances (J. Riley, L. Wojcik, W. Huang, S. Xu, S. Kuhmann, et al., unpublished data). In another study, two maraviroc-resistant isolates derived from the primary isolates RU570 and CC1/85 were generated in PBMCs [21]. The resistance of both viruses was associated with V3 changes, but even when the same CC1/85 isolate was used in different experiments, different genetic changes conferred resistance [21]. After 20 passages without maraviroc, the resistant isolates partially (CC1/85) or completely (RU570) reverted to sensitivity [21]. Overall, HIV-1 can follow different genetic routes to resist small molecule CCR5 inhibitors, pathways perhaps associated with varying degrees of fitness loss.

### The Effect of AD101 on the Fitness of AD101-Resistant Clones

How resistance to small molecule CCR5 inhibitors is manifested can be both assay- and cell type–dependent [20]. When the replication-competent Env-chimera CC101.19 cl.7 was tested in a PBMC-based replication assay, the AD101-related compound vicriviroc caused no inhibition, but rather modestly but consistently enhanced p24 production. In contrast, when the same *env* gene was used in a single round, Env-pseudotype assay involving PBMCs, vicriviroc was partially inhibitory, the extent of inhibition plateauing at ~25%. When the same Env-pseudotype virus was studied in U87-CD4/CCR5 cells, an inhibition plateau was again observed, but now at ~90% inhibition. We have concluded that CC101.19 cl.7 can enter cells by using both the inhibitor-bound and inhibitor-free forms of CCR5, and that the height of the plateau, when it occurs, is a measure of the relative efficiency with which the two CCR5 configurations are used, a parameter influenced by the cell type [20]. Similar conclusions have been drawn regarding maraviroc resistance [21].

To gain further insights, we investigated whether entry via the AD101-bound form of CCR5 influences the fitness of the AD101-resistant clones, using a saturating AD101 concentration (20 μM) to ensure that no free receptor remained on the target cells. All three CC101.19-derived clones were fitter in the presence of AD101 than they were in its absence (Figure 7; Table 2). This is mechanistically informative, because HIV-1 entry is not enhanced by vicriviroc or AD101 in single-round assays ([20]; unpublished data). As the fitness assays involve 14 d of replication in PBMC culture, it seems likely that the fitness increase conferred by AD101, like the replication enhancement caused by vicriviroc, only arises during multiple replication cycles; we are now investigating why this occurs. One possible factor is that CCR5 ligands like AD101 and vicriviroc can upregulate both CCR5 and its chemokine ligands MIP-1β and RANTES in PBMC cultures, events irrelevant for single-cycle entry assays [20]. The interplay between the various CCR5 ligands (chemokines, small molecule inhibitors, and gp120) during a multi-cycle replication process is likely to be complex.

### Implications for the Clinical Use of CCR5 Inhibitors to Treat HIV-1 Infection

Two small molecule CCR5 inhibitors, maraviroc and vicriviroc, are now in advanced clinical trials as therapeutics, and cause significant (~1.5 log_10_) viral load reductions [38,39]. As with all HIV-1 therapies, resistance development must be anticipated [15,16]. The existing classes of antiretrovirals sometimes provide continued therapeutic benefit even when resistance arises, and the drug-sensitive phenotype reemerges when the selecting drug is withdrawn. These events are hallmarks of a fitness cost to resistance, suggesting that fitness decreases measured in vitro do have clinical relevance [2,14,27].

There are real limitations to in vitro assays when predicting what might happen to HIV-1 in vivo, particularly for studies involving the envelope glycoproteins [16]. These proteins face multiple evolutionary pressures in vivo, conferred by neutralizing antibodies, changes in the numbers and types of target cells, alterations in the number and nature of their co-receptors, and the presence of both natural (chemokine) and unnatural (drugs, when present) co-receptor ligands. Most of these selection pressures are absent in vitro, or else present as uncontrolled variables (e.g., production of chemokine ligands in PBMC cultures noted above). There are changes in the in vitro sensitivity of CCR5 inhibitor-resistant viruses to certain neutralizing monoclonal antibodies (P. Pugach and J. Moore, unpublished data), but here again the in vivo situation will be more complex.

If, however, our central observation—that some CCR5 inhibitor-resistant viruses are no less fit than parental strains—is directly relevant to what might happen in vivo, the implication is that such viruses would persist for prolonged periods after CCR5 inhibitor therapy is discontinued. Although no inhibitor would then be present to occupy CCR5, the resistant viruses are not “drug-dependent”, but can use the free receptor. One theoretical concern is that the alteration in how resistant viruses utilize CCR5 could enable them to use CCR5 conformational variants differently or more efficiently, opening up additional target cells to infection. HIV-1 variants with higher CCR5 affinities and lower sensitivities to CCR5 ligands can arise naturally during HIV-1 infection, because of the drop in the average levels of CCR5 available on target cells [40–44]; CCR5 inhibitor-resistant variants might, in principle, have a similar advantage over wild-type viruses under certain conditions. Nonetheless, naturally occurring viruses with complete resistance to CCR5 inhibitors have rarely been observed ex vivo or in vivo. Thus, whatever replication advantage might be conferred by Env configuration changes allowing use of the CCR5-inhibitor complex, other counter-selection pressures presumably prevent this from occurring naturally. Ex vivo analyses of viruses derived from long-term clinical studies of CCR5 inhibitors, together with the general experience gained from these trials, might clarify some of the above issues.

## Materials and Methods

### Reagents.

AD101 (SCH-350581) was provided by Julie Strizki (Schering-Plough Research Institute, Kenilworth, New Jersey, United States). The small molecule CXCR4 inhibitor AMD3100 was obtained from the National Institutes of Health (NIH) AIDS Research and Reference Reagent Program.

### Viruses.

Viruses used in the fitness studies include the parental R5 isolate (CC1/85), the AD101-resistant isolate (CC101.19), and three clonal, NL4–3/Env-chimeric infectious viruses derived from each of these isolates (Table 1). Mutants of the one of the CC1/85 clones with varying degrees of resistance include a virus with one amino acid change in V3 (H308P) conferring partial AD101 resistance, and one with all four V3 changes (K305R, H308P, A316V, G321E) necessary and sufficient for full resistance [17]; these clonal viruses are designated “CC1/85 cl.7 (HP)” and “CC1/85 cl.7 (V3),” respectively. Their construction and properties have been described [17,20]. A clone from the passage 20 control isolate (CCcon.20 cl.11) [36] was also studied. Clonal proviruses containing the mutated *vif* sequence were constructed as below. Infectious virus stocks were prepared by transient transfection of 293T cells with pNL4–3/*env* plasmids using Lipofectamine 2000 (Invitrogen, http://www.invitrogen.com) according to the manufacturer's instructions, as described [17,20,36]. Stocks of the CC1/85, CC101.19, and CC101.22R9 isolates were prepared as described [19]. All infectious stocks were stored in aliquots at −80 °C. The titers (50% tissue culture infectious dose [TCID_50_]) of all stocks were determined in PBMC culture by standard methods [45].

### Cell culture and extended virus passage.

CD8-depleted PBMC cultures were purified and stimulated as described [17,18,20]. PBMCs from between two to four random donors were mixed in equal proportions to reduce donor-dependent effects on viral replication. After stimulation, PBMCs were maintained in lymphocyte medium (RPMI 1640 + 10% FBS + 2 mM L-glutamine + 100 U/ml IL-2). The CC101.22R9 isolate [19] was returned to culture for 11 additional passages in activated PBMC culture without AD101. Passages were performed weekly by adding a 5-ml aliquot of culture supernatant and cells from the previous passage to 15 ml of freshly stimulated PBMCs at 2 × 10^6^ cells/ml. The remaining supernatant was filtered and frozen in 1-ml aliquots at −80 °C for drug sensitivity testing.

### Viral replication kinetics.

To determine the growth kinetics, 100 TCID_50_ of the test virus were used to inoculate 2 × 10^5^ cells in 200 μl of lymphocyte medium (MOI = 0.0005) in replicate wells of a 96-well plate. After incubation at 37 °C for the indicated times, duplicate wells were harvested and virus was inactivated by addition of 1% Empigen BB detergent (Sigma-Aldrich, http://www.sigmaaldrich.com). All samples from a single experiment were tested simultaneously for p24 antigen using an in-house ELISA [46]. Replication rates were determined by plotting the increase in p24 antigen over time. The sensitivities of isolates CC1/85, CC101.19, CC101.22R9, CC101.22R10, CC101.22R15, and CC101.22R20 to inhibition by AD101 were assayed as described previously [17]. Briefly, PBMC cultures were infected in 96-well plates, as described above, in the presence of varying AD101 concentrations, then p24 concentrations were measured after 7 d.

### Construction of pNL4–3 vif tracking vector and real-time PCR standard.

The first step in the construction of the mutated pNL4–3 *vif* tracking vector, pNL4–3 vifY, involved subcloning of the region of pNL4–3 located between the SphI (position 1448) and EcoRI (position 5744) restriction sites into pNEB193 (New England Biolabs, http://www.neb.com). The resulting pNEB-NL4–3 was subjected to site-directed mutagenesis using the Stratagene (http://www.stratagene.com) Quickchange I kit and the following primers, as specified by the manufacturer: HIV Vif Sense (5′-CCA TAG AAT GGA GGA AAA AGA GAT ATA GC-3′), HIV Vif AntiS (5′-GTT GCA GAA TTC TTA TTA TGG C-3′), HIV VifY B sense (5′-AGC TTG CAA TAT CTA GCG TTG GCA GCA TTA ATA AAA CCA AAA CAG-3′), and HIV VifY B AntiS (5′-CAA CGA TAG ATA TTG CAA GCT TCC TAC CTT GTT ATG TCC TGC-3′) to form pNEB-NL4–3-vifY. The mutated SphI to EcoRI fragment was sub-cloned back into pNL4–3 to form pNL4–3-vifY and sequenced using the Vif Seq S primer (5′-TGG CAA GTA GAC AGG ATG AGG A-3′). To construct the CC1/85 cl.7 and CC101.19 cl.7 reference proviruses with the vifY allele, the *env* sequence was removed from the appropriate pNL4–3-*env* plasmid using the EcoRI and XhoI restriction sites, then ligated into the corresponding sites in pNL4–3-vifY. We verified that the vifY sequence in *vif* did not revert to the wild-type (vifX) form under the conditions of growth-competition assays (unpublished data).

The QPCR standard was created by first cloning the wild-type NL4–3 *vif* (VifX) sequence into the pCR2.1 TOPO vector (Invitrogen), using the HIV Vif Sense and HIV Vif AntiS primers described above, to form pCR2.1-vifX. The wild-type *vif* sequence was then sub-cloned from pCR2.1-vifX into pNEB-NL4–3-vifY using the EcoRI restriction site. The plasmid, pVifStd, contained tandem copies of both the vifX and vifY alleles. The concentration of this standard was quantified by UV absorbance spectrophotometry. Ten-fold serial dilutions were used as templates to generate standard curves in the real-time TaqMan PCR assays.

### Growth-competition assays.

These were performed in 48-well plates seeded with 2 × 10^6^ stimulated PBMCs in 0.8 ml. The two viruses under evaluation were added to the target cells at individual MOIs of 0.0001 or 0.0005, which are generally accepted to be low enough to prevent recombination [35]. Three competitions were established for each pair at each MOI, using different ratios of the input viruses (1:1, 1:10, and 10:1). When appropriate, inhibitors were incubated with target cells for 1 h before virus addition. To limit the inherent variability of PBMC replication assays, each data point was derived from duplicate cultures on the same plate, and all experiments were performed at least thrice. Additional controls included in each experiment entailed mono-infections of PBMCs with each virus separately at different MOIs (low or high) in the presence or absence of various AD101 concentrations. Competition cultures involving X4 and R5 viruses were maintained for 10 and 14 d, respectively. The cells were then harvested, washed once with phosphate-buffered saline (PBS), and pelleted for DNA extraction using the QIAamp DNA Blood Mini Kit, according to the manufacturer's instructions (Qiagen, http://www.qiagen.com).

### TaqMan QPCR assay.

Competition experiments were analyzed using a multiplexed TaqMan PCR assay. First, 5 μl of extracted DNA were subjected to a brief, external PCR amplification reaction in a final volume of 25 μl, using the Vif subtype B S (5′-TGG CAG GTG ATG ATT GTG TGG CA-3′) and Vif subtype B AntiS (5′-GGT CTT CTG GGG CTT GTT CCA TCT-3′) primers and AccuPrime SuperMix II, as specified by the manufacturer (Invitrogen). DNA amplifications were performed under the following cycling conditions: 1 cycle at 94 °C for 2 min; 10 cycles at 94 °C for 30 s, 55 °C for 30 s, and 68 °C for 45 s; and 1 cycle at 68 °C for 2 min. The reaction product was then purified using the QIAquick PCR Purification Kit (Qiagen).

The TaqMan assay utilizes probes to differentiate between the two forms of the NL4–3 backbone (vifX and vifY). The probes are labeled with different fluorescent markers at their 5′-ends. In solution, prior to binding of the primers and probes to their target sequences, and during annealing, the quenching agent on the opposite end of the probes dampens the fluorophores' signals. However, during the PCR extension step, the probe becomes vulnerable to the 5′ to 3′ exonuclease activity of Taq DNA polymerase; freed from proximity to the quencher, the fluorophore can now emit a fluorescence signal that is amplified logarithmically by successive rounds of PCR and monitored in real-time. The number of PCR cycles required to reach a given threshold fluorescence value is inversely proportional to the logarithm of the input quantity of dsDNA containing the probe sequence. By quantifying the fluorescence output from both probes, each competing viral genome can be quantified simultaneously from a single sample. In the assay, the Brilliant QPCR Master Mix (Stratagene) was used with the primers Vif beta S (5′-AGT TAG TCC TAG GTG TGA-3′) and Vif beta AS (5′-TCC ATC TGT CCT CTG TCA-3′) and the reference dye ROX, according to the manufacturer's specifications. Included in the reaction were the vifX and the vifY probes (sequences are provided in Figure 2A) that were labeled with Cy5 and Black Hole Quencher 2 (vifX), and with FAM and Black Hole Quencher 1 (vifY) at the 5′- and 3′-ends, respectively. The reactions were run on a Stratagene Mx4000 machine using the cycling conditions: 95 °C for 10 min, then 95 °C for 30 s and 55 °C for 1 min for 40 cycles. All reactions were performed in triplicate, including the seven serial dilutions of the standard DNA template, (ranging from 5 × 10^7^ to 5 × 10^1^ copies), as well as a negative control (no-template). The mean values of the measured numbers of copies per reaction were then determined using the Stratagene Mx4000 software version 4.20, and used for further analysis. The slope and correlation coefficient of each standard curve were calculated based on the average threshold cycle (*C_T_*) values measured in triplicate for each dilution point. The PCR efficiency, *E,* was computed as (10^−1/*s*^ − 1) × 100%, where *s* is the slope of the generated standard curve.

### Calculation of viral fitness.

Fitness differences (W_D_) were calculated for each clonal virus based on its relative production in head-to-head competition, as previously described [3]. Initially, the copy numbers per QPCR reaction of the vifX and vifY alleles in each infection well were determined from the triplicate QPCR reactions for each infection condition; copy numbers for the duplicate infection conditions were then averaged. The copy numbers of the 10:1 and 1:10 infections were weighted by their initial proportions in the inoculum, and the weighted copy numbers from the three conditions (1:1, 10:1, and 1:10) were averaged to give a single copy number per experimental condition (*n*_vifX_ and *n*_vifY_). The proportion of each virus at each condition (*w*_vifX_ and *w*_vifY_) was then determined from the ratio of the copy number of that virus (*n*_vifX_ or *n*_vifY_) to the total copy number (*w*_vifX_ = *n*_vifX_ */*[*n*_vifX_ + *n*_vifY_] and *w*_vifY_ = *n*_vifY_ */*[*n*_vifX_ + *n*_vifY_]). The fitness difference (W_D_) for an individual experiment was calculated by determining the ratio of these proportions, always comparing the test vifX virus to the reference vifY virus (W_D_ = *w*_vifX_/*w*_vifY_). Thus, when W_D_ > 1, the vifX virus has won the competition, and when W_D_ < 1, the vifY virus has prevailed. When the W_D_ value is based on more than one experiment, the proportions (*w*_vifx_ and *w*_vifY_) from each experiment were averaged before calculating the W_D_ value, to avoid giving undue weight to any single experiment.

For W_D_ values calculated from single experiments where the vifX and vifY viruses had the same *env* gene (Figure 2D), the greatest deviation from the expected W_D_ value of 1 was 0.35. Thus, we conservatively conclude that W_D_ values >3 or <0.33 indicate that two viruses in direct competition differ significantly in their replication capacities. If the minimum copy numbers per QPCR reaction produced in mono-infected cultures was ~5 × 10^4^ (it was frequently higher) and the minimum copy numbers that could be detected is 50 (it was probably lower), then we would be able to detect fitness differences of up to 1,000-fold. Hence, using these conservative estimates, we conclude that the working range for the assay is W_D_ values of 0.0001 to 1,000.

## Supporting Information

### Accession Numbers

The GenBank (http://www.ncbi.nlm.nih.gov/Genbank/index.html) accession numbers for CC1/85- and CC101.19-derived *env* clones used in this study are CCcon.20 cl. 11 (AY357537), CC1/85 cl. 6 (AY357338), CC1/85 cl.7 (AY357341), CC1/85 cl.8 (AY357344), CC101.19 cl.3 (AY357466), CC101.19 cl.7 (AY357465), and CC101.19 cl.15 (AY357468). The GenBank accession number of the plasmid pNL4–3 containing the NL4–3 provirus is AF324493.

## Abbreviations

HIV-1: human immunodeficiency virus type 1
MOI: multiplicity of infection
PBMC: peripheral blood mononuclear cell
QPCR: quantitative PCR
